# Changes of plasmalogen phospholipid levels during differentiation of induced pluripotent stem cells 409B2 to endothelial phenotype cells

**DOI:** 10.1038/s41598-017-09980-x

**Published:** 2017-08-24

**Authors:** Yusuke Nakamura, Yasuo Shimizu, Yasuhiro Horibata, Rinna Tei, Ryosuke Koike, Meitetsu Masawa, Taiji Watanabe, Taichi Shiobara, Ryo Arai, Kazuyuki Chibana, Akihiro Takemasa, Hiroyuki Sugimoto, Yoshiki Ishii

**Affiliations:** 1 Department of Pulmonary Medicine and Clinical Immunology, Dokkyo Medical University School of Medicine, 880 Kitakobayashi, Mibu, Tochigi 321-0293 Japan; 2 Department of Biochemistry, Dokkyo Medical University School of Medicine, 880 Kitakobayashi, Mibu, Tochigi 321-0293 Japan

## Abstract

Endothelial cells (EC) are involved in regulating several aspects of lipid metabolism, with recent research revealing the clinicopathological significance of interactions between EC and lipids. Induced pluripotent stem cells (iPSC) have various possible medical uses, so understanding the metabolism of these cells is important. In this study, endothelial phenotype cells generated from human iPSC formed cell networks in co-culture with fibroblasts. Changes of plasmalogen lipids and sphingomyelins in endothelial phenotype cells generated from human iPSC were investigated by reverse-phase ultra-high-pressure liquid chromatography mass spectrometry (UHPLC-MS/MS) analysis. The levels of plasmalogen phosphatidylethanolamines (38:5) and (38:4) increased during differentiation of EC, while sphingomyelin levels decreased transiently. These changes of plasmalogen lipids and sphingomyelins may have physiological significance for EC and could be used as markers of differentiation.

## Introduction

Endothelial cells (EC) are multifunctional cells with a role in regulating coagulation and fibrinolysis, vascular tone, angiogenesis, and inflammatory reactions, and EC dysfunction is involved in the pathophysiology of various diseases^1, 2^. With regard to respiratory diseases, vasodilation contributes to limitation of airflow in asthma^3–5^, and EC dysfunction is related to smoking–induced chronic obstructive pulmonary disease (COPD)^6^. In patients with cardiovascular disease, EC injury or dysfunction is the first step toward the development of atherosclerosis^7, 8^.

Stem cells are expected to have medical applications in cell therapy for the above-mentioned diseases. EC derived from mouse or human induced pluripotent stem cells (iPSC) were reported in 2008 and 2009 by the same group^9, 10^, and it has been shown that cells positive for Flk1 (also known as KDR/vascular endothelial growth factor receptor 2, VEGFR2) and VE-cadherin differentiate into EC^11^. Engineering of the pulmonary vasculature has been performed in decellularized rat and human lungs by using EC and perivascular cells derived from iPSC^12^. Furthermore, patient-specific EC derived from iPSC have demonstrated a protective effect against pulmonary hypertension in familial pulmonary arterial hypertension with bone morphogenetic protein receptor type 2 (BMPR2) mutation^13^. Moreover, EC derived from iPSC demonstrate endothelial functionality *in vitro* and have been shown to promote neovascularization and enhance restoration of blood flow in animal models of myocardial infarction and peripheral arterial disease^14^. Thus, EC derived from iPSC have the potential for various medical applications^15, 16^, but elucidation of the biological features of these cells is required before clinical use can be attempted.

Glycerophospholipids are components of lipid bilayer membranes that are classified into three groups based on *sn-1* linkage. Diacyl glycerophospholipids have a diester linkage at the *sn-1* and *sn-2* positions, plasmanyl glycerophospholipids have an ether linkage at the *sn-1* position, and plasmalogens (also known as plasmenyl glycerophospholipids) contain a vinyl ether linkage at the *sn-1* position. Typical plasmalogens have C16:0, C18:0, or C18:1 at the *sn-1* and a polyunsaturated fatty acid at the *sn-2* position^17, 18^. The most common plasmalogens in total phospholipids are plasmalogen phosphatidylethanolamines (pPE: 5–22%) and plasmalogen phosphatidylcholines (pPC: 0.8–22%), whereas serine or inositol plasmalogens are rare^17^. However, the levels of these plasmalogens vary among cells or tissues^17^. The antioxidant effect of endogenous plasmalogens has been examined in detail, and plasmalogens have been shown to protect EC from hypoxic stress and reactive oxygen species (ROS)^19^. Reduced levels of plasmalogens have been reported in ageing, dyslipidemia, Alzheimer’s disease, smoking, and coronary artery disease^20–24^.

Sphingomyelins (SM) are basic components of cell membranes and a major source of ceramides, with production being regulated by SM synthases (SMS) and sphingomyelinases (SMases)^25^. High plasma SM levels were reported to predict more rapid annual progression of pulmonary emphysema^26^. Inhibition of SMS in EC was reported to attenuate lipopolysaccaride (LPS)-induced lung injury in animals through enhancement of the EC barrier by reducing loss of SM from lipid rafts in the cell membrane^27^. Elevated plasma SM levels are a prognostic marker in acute coronary syndrome^28^, and overexpression of SMS is suggested to be related to EC dysfunction, a decrease of CD34/KDR-positive endothelial progenitor cells, and development of atherosclerosis in ApoE knock-out mice^29^. Thus, plasmalogens and SM play an important role in EC function and their plasma levels reflect various disease states. Therefore, elucidating the behavior of plasmalogens and SM in iPSC-derived EC during differentiation could be helpful for evaluating the potential of medical applications of EC generated from iPSC.

In the present study, endothelial phenotype cells were generated from human iPSC, and the levels of pPE and pPC (major components of plasmalogen lipids) and SM were examined in iPSC-derived EC by liquid chromatography-mass spectrometry (LC-MS). Levels of these lipids were evaluated during differentiation of iPSC to endothelial phenotype cells and were compared with the levels in established cell lines of EC and fibroblasts.

## Results

### Induction and purification of endothelial cells

**Figure 1:**
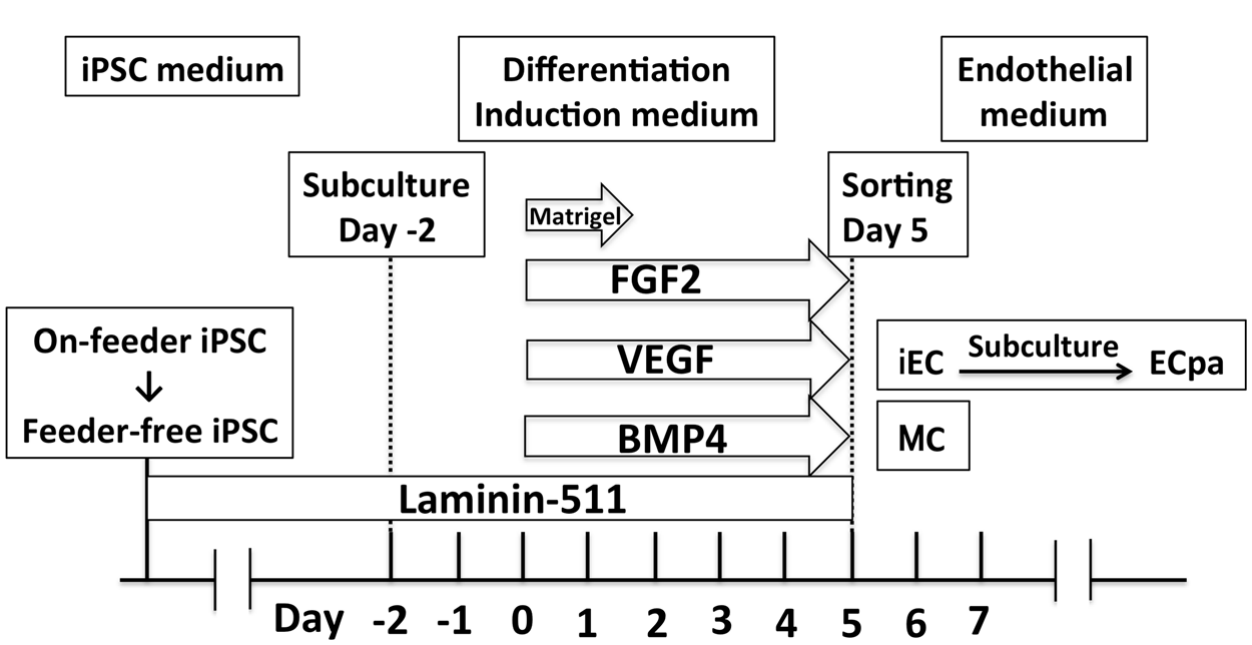
Induction of EC from iPSC. iPSC were transferred from on-feeder culture to feeder-free culture. After passaging on day −2, the cells were stimulated on day 0 with fibroblast growth factor 2 (FGF2: 10 ng/mL), vascular endothelial growth factor A-165 (VEGF: 50 ng/mL), bone morphogenetic protein 4 (BMP4: 2 ng/mL), and Matrigel (1/100 v/v, only on day 0). The medium was changed every 2 days and cells were sorted into iEC and MC on day 5. iEC were cultured for several more passages to obtain ECpa, which were used in the network formation assay.

Induction of EC from iPSC was performed as shown in Fig. 1. Pluripotency of feeder-free iPSC was confirmed by SSEA-4 immunofluorescence staining and the alkaline phosphatase assay (Supplementary Figure S1). On day 5 of culture, cells were sorted by a FACSAria^®^ FACS with KDR and VE-cadherin fluorescent labeling. Mesodermal cells (MC) were defined as KDR^+^/VE-cadherin^−^ cells, while EC were KDR^+^/VE-cadherin^+^ cells. The mean percentage of MC differentiated from iPSC was 18.0 ± 1.2% on day 4, 11.0 ± 5.5% on day 5, and 20.6 ± 2.0% on day 6. In addition, the mean percentage of EC sorted from the cultures (iEC) was 11.9 ± 2.4% on day 4, 10.9 ± 2.6% on day 5, and 7.4 ± 0.5% on day 6 (Supplementary Figure S2a,b). The initial morphological changes as iPSC underwent differentiation into iEC were observed on day 3 or 4. Typical iPSC are characterized by large nuclei and scanty cytoplasm, but these features were modified on days 3 to 4 (Supplementary Figure S2c).

### Purification of endothelial cells derived from iPSC and functional assessment

**Figure 2:**
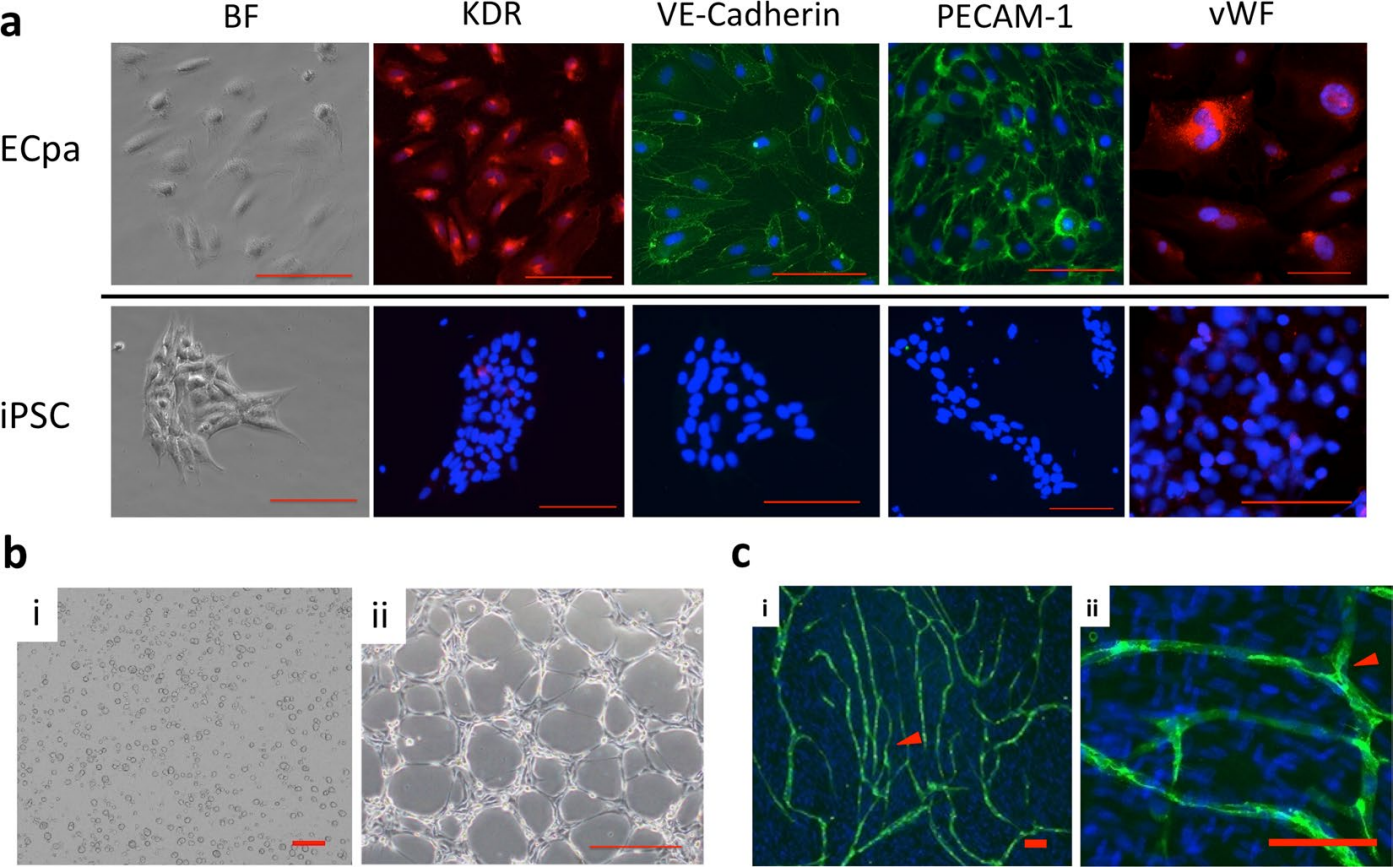
Assessment of induced EC. Immunofluorescence staining of iPSC and ECpa (**a**). iPSC were not positive for PECAM-1, VE-cadherin, KDR and vWF (EC markers), whereas nearly 100% of ECpa were positive for PECAM-1, VE-cadherin, KDR and vWF. (BF = bright field) (*Scale bar* = 100 *μm*). Network (tube) formation assay (**b**). ECpa were cultured in 12-well plates coated with extracellular matrix (ECM). i: 0 hours of incubation. ii: 4 hours of incubation. Network formation was observed at 4 hr. (*Scale bar* = 100 *μm*). Co-culture network (tube) formation assay (**c**). When HFL-1 cells and ECpa were co-cultured, networks were formed sterically between HFL-1 cells. i: Fluorescent immunostaining: PECAM-1 (green), DAPI (blue). ii: Magnified view of fluorescent immunostaining. Arrowhead indicated the tube formation by ECpa. (*Scale bar* = 100 *μm*).

The iEC but not MC were subcultured at least twice (ECpa), immunostaining was done with anti-KDR, anti-VE-cadherin, anti-PECAM-1 and anti-vWF antibodies. Most ECpa (nearly 100%) were positive for KDR, VE-cadherin, PECAM-1 and vWF while iPSC were negative for these markers (Fig. 2a). The expression of vWF mRNA measured by quantitative PCR was significantly higher in iEC or ECpa compared with iPSC. Immunostaining also showed that iEC were positive for vWF antibody (Supplementary Figure S3). To assess the functional characteristics of ECpa, the tube formation assay was performed. ECpa were round single cells shortly after seeding (Fig. 2bi), but started to form networks after incubation for 2 hours. Network formation was maximal around 4 to 12 hours (Fig. 2bii), after which agglomeration of the networks occurred. To evaluate tube-forming capacity in co-culture, ECpa were co-cultured with human fetal lung fibroblast-1 (HFL-1) cells, followed by immunostaining with PECAM-1 (green) to detect ECpa and DAPI (blue) for nuclei. Network formation by ECpa was confirmed by detection of PECAM-1-positive networks in the co-cultures (Fig. 2ci). At a higher magnification, fluorescent immunostaining showed that networks were formed sterically between HFL-1 cells (Fig. 2cii). Without immunostaining, the ECpa networks were difficult to distinguish from HFL-1 cells (Supplementary Figure S4). ECpa purification efficiency was confirmed by VE-Cadherin/PECAM-1 double positive ratio. Nearly 90% of ECpa was VE-cadherin^+^/PECAM-1^+^ positive (Supplementary Figure S5).

### Results of LC-MS/MS

**Figure 3:**
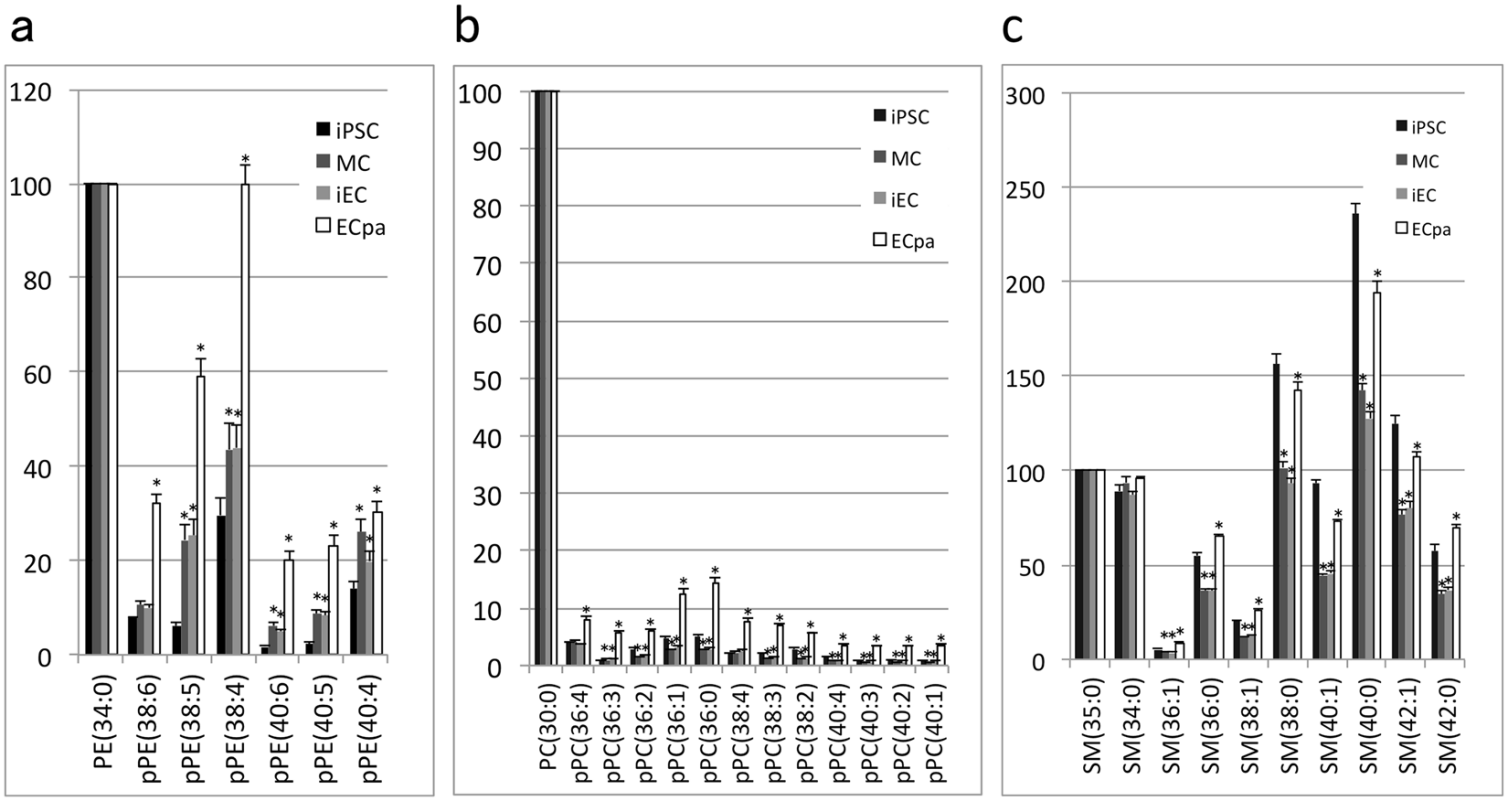
Changes of phospholipids detected by LC-MS/MS during differentiation of iPSC to ECpa. Levels of pPE (**a**), pPC (**b**), and SM (**c**) during differentiation of iPSC to ECpa were examined. PE (34:0), PC (30:0), and SM (35:0) were used as internal standards, with 1 μg being added to 100 μg of protein solution. (*p < 0.05 compared with iPSC).

**Figure 4:**
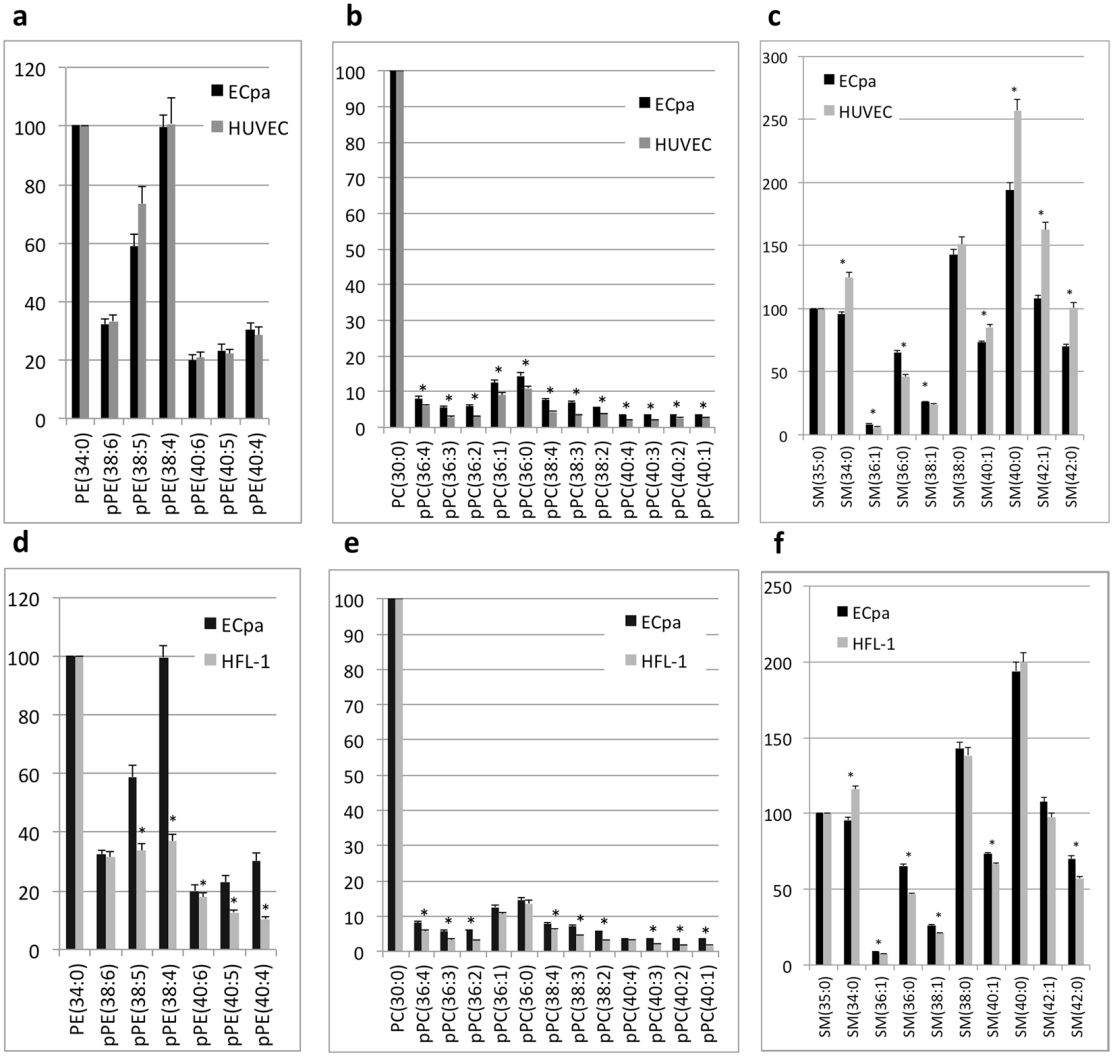
Composition of pPE, pPC, and SM in ECpa, HUVEC and HFL-1 cells determined by LC-MS/MS. Levels of pPE, pPC, and SM in ECpa were compared with HUVEC (**a**–**c**) and HFL-1 (**d**–**f**). PE (34:0), PC (30:0), and SM (35:0) were used as internal standards, with 1 μg being added to 100 μg of protein solution. (*p < 0.05).

Lipids were collected from iPSC in feeder-free culture on day −2 before induction of differentiation. MC and iEC were sorted on day 5 (KDR^+^/VE-cadherin^−^ cells were defined as MC and KDR^+^/VE-cadherin^+^ cells were defined as iEC). The contents of pPE, pPC, and SM were measured in iPSC, MC, iEC, and ECpa. Internal standards for pPE, pPC, and SM were phosphatidylethanolamine (PE) (34:0), phosphatidylcholine (PC) (30:0), and SM (35:0), respectively. These standards (1 μg) were added to 100 μg of protein solution at the purification step. In Figs 3 and 4, ratios to the internal standards are shown on the vertical axis. Levels of pPE(38:5), pPE(38:4), pPE(40:6), pPE(40:5), and pPE(40:4) were significantly increased in MC, iEC, and ECpa compared with iPSC, and were especially high in ECpa. The pPE (38:6) level was not significantly higher in MC and iEC than in iPSC, but increased markedly after passaging as shown in ECpa (Fig. 3a). Levels of pPC (36:4) to pPC (40:1) were not significantly different among iPSC, MC, and iEC, but were significantly higher in ECpa compared with the other cells (Fig. 3b). On the other hand, SM showed a transient decrease during differentiation of MC and iEC, and subsequently increased in ECpa (Fig. 3c).

Since pPE and pPC were significantly increased in ECpa, the levels of these lipids were compared between ECpa and HUVEC, which are regarded as endothelial phenotype cells. Levels of pPE components were not significantly different between ECpa and HUVEC (Fig. 4a), but levels of pPC components were significantly lower in HUVEC (Fig. 4b). SM showed a transient decrease during differentiation, so SM components were also compared between ECpa and HUVEC. SM (36:1) and SM (36:0) were higher in ECpa than HUVEC, but other SM components were low, except for SM (38:0) (Fig. 4c). Since fibroblasts are usually derived from MC, the pPE, pPC, and SM levels were also measured in HFL-1 cells, which are fibroblasts. It was found that the levels of pPE (38:5), pPE (38:4), pPE (40:6), pPE (40:5), and pPE (40:4) were significantly higher in ECpa than HFL-1 cells, but pPE(38:6) was not significantly different (Fig. 4d). While pPC levels were significantly higher in ECpa than in HFL-1 cells, the differences were smaller than those observed for pPE (Fig. 4e). In addition, the levels of SM(36:1), SM(36:0), SM(38:1), SM(40:1), and SM(42:0) were significantly higher in ECpa than in HFL-1 cells, but SM(34:0) was lower in ECpa cells (Fig. 4f). Culture medium were needed to be preparing for each cell lines because of keeping their phenotype, such as pluripotency, EC or fibroblasts. Since culture medium would affect the cellular phospholipid contents, levels of pPE, pPC and SM in culture medium were measured. Most of pPE and pPC levels in medium were low of under detection levels, however SM (34:0) was confirmed high in F12K (Supplementary Figure S6).

## Discussion

In the present study, human EC (characterized as KDR^+^ PECAM-1^+^VE-cadherin^+^VWF^+^ cells) generated from human iPSC showed angiogenic potential and ECpa also formed cellular networks in co-culture with fibroblasts. During differentiation from iPSC to EC, the pPE level increased significantly. In addition, pPE levels were similar in ECpa and HUVEC, indicating that pPE is a potential marker for endothelial phenotype cells.

Several methods of inducing EC from iPSC have been reported^30, 31^. Kohler *et al*. developed a simple and excellent method for generating functional murine EC from murine iPSC^30^, and we modified their method for use in the present study. In order to increase the number of attached iPSC in feeder-free dishes, we used laminin-511-E8 (iMatrix511^®^) to coat the dishes instead of type IV collagen before seeding iPSC, while Matrigel^®^ was used to reduce the number of cells becoming detached from the culture dishes at the first step of induction, which was addition of cytokines. Laminin-511-E8 and Matrigel^®^ contain several matrixes and collagens, and have been reported to provide support or increase the efficiency of iPSC adhesion and growth^32^. Since we could not obtain enough EC for subculture by the original method^30^, we modified it for this study (Supplementary Figure S7). The reasons for the lower efficiency of inducing EC than previously reported are unknown, but technical matters or use of different cell lines might have had an influence. Previous report^30^ used the pre-coated dish of unknown concentration of type IV collagen coating. In present study, type IV collagen was tested at the similar concentration to Laminin-511-E8. This also might have had an influence. However, the cells derived from iPSC by our modified method were confirmed to have the characteristics of EC by immunofluorescence and functional assessment, as reported previously^30, 33^. After these EC were passaged (ECpa), the cells formed networks in co-culture with HFL-1 cells. HFL-1 cells are known to be fibroblasts that undergo transformation to the contractile phenotype of myofibroblasts in response to certain stimuli^34^. The existence of myofibroblasts in interstitial tissue has been reported in pulmonary fibrosis, and myofibroblasts contribute to the development of fibrosis^35^. In order to generate EC from the iPSC of patients with diseases such as pulmonary fibrosis, this co-culture system could be used as a tool for analysis of the influence of various stimuli in a pulmonary fibrosis model.

Plasmalogen phospholipids have been identified in murine embryonic stem cells (ESC), iPSC, and fibroblasts. It was reported that the cellular content of pPE (34:1), pPE (34:2), and pPE (36:2) was higher in fibroblasts than in ESC or iPSC, while the level of pPE (34:2) was lower in fibroblasts than in ESC or iPSC. In addition, the pPC (34:1) content was higher in fibroblasts than in ESC and iPSC, while the pPC (36:4) content was lower in fibroblasts than in ESC and iPSC^36^. The present study showed that the levels of pPE (38:6), pPE (38:5), pPE (38:4), pPE (40:6), pPE (40:5), and pPE (40:4) all increased significantly during differentiation of iPSC to iEC and after passaging of iEC (ECpa). Most of the pPCs, especially pPC (36:3), pPC (36:2), pPC (36:1), pPC (36:0), pPC (38:4), pPC (38:3), and pPC (38:2), showed similar changes to those of the pPEs. These findings suggest that most plasmalogens might increase during differentiation to mature cells such as fibroblasts and EC. Since it is known that pPE is metabolized to pPC, the increase of pPC might be secondary to elevation of pPE^37^.

We compared plasmalogen levels in ECpa with those in HUVEC (endothelial cells) and HFL-1 cells (fibroblasts). Detection of pPE and pPC in HUVEC was reported previously^38^. The pPE levels were not different between ECpa and HUVEC in present study. When the levels of pPE (38:5) and pPE (38:4) were compared between ECpa and HFL-1 cells, both pPE (38:5) and pPE (38:4) were significantly higher in ECpa cells. In short, these results investigations revealed that pPE (38:5) and pPE (38:4) levels showed a marked increase during culture of iEC to ECpa, with pPE (38:5) and pPE (38:4) levels also being increased in endothelial cells and higher than in HFL-1 cells. Taken together, these results suggest that pPE (38:5) and pPE (38:4) could be markers for EC phenotype acquisition.

Metabolic signatures and markers are important when considering medical applications of cells. EC generated from iPSC are usually identified from cell surface markers (KDR^+^ PECAM-1^+^VE-cadherin^+^) and from the ability to promote angiogenesis *in vitro* or *in vivo* ^14, 30^. Previous studies have shed some light on the dynamic barrier properties (influenced by multiple vascular permeability factors) of EC generated from iPSC^39^, as well as transcriptional and metabolic changes during maturation of EC differentiated from iPSC^40^. Plasmalogens play an important role in the response of EC to disease, and supplementation of plasmalogens was reported to support retinal vascular development^41^. Therefore, when considering the application of generated EC for cell therapy, it may be useful to evaluate the levels of plasmalogens such as pPE (38:5) and pPE (38:4). Plasmalogens contain a vinyl ether linkage at the *sn-1* position and polyunsaturated fatty acids at *sn-2* ^17^. The fatty acids at *sn-1* or *sn-2* could not be determined in present study. However, it is known that the *sn-1* position is commonly occupied by a C16:0 or C18:0 or C18:1 fatty acid and the *sn-2* position is commonly occupied by arachidonic acid (AA 20:4) or docosahexaenoic acid (DHA C22:6)^37^. Therefore, pPE (38:5) and pPE (38:4) are possibly composed of the combination of C18:0 or C18:1 and AA 20:4, respectively. When pPC levels were compared between ECpa and HUVEC or HFL-1 cells, large differences like those of pPE were not observed, suggesting that there might be differences in the metabolism of pPE and pPC. Taken together, pPE (38:5) and pPE (38:4) could be markers for EC.

The pathophysiological importance of pPE and pPC has been reported. Increased serum levels of pPE (38:5) and pPE (38:4) are associated with a higher risk of myocardial infarction^42^. High-density lipoprotein cholesterol (HDL-C) contributes to plaque regression^43^, and the levels of pPE (38:6), pPE (40:6), and pPC (36:4) were significantly increased in HDL-C compared to low-density lipoprotein cholesterol (LDL-C) by statin therapy in patients with dyslipidemia^44^. In addition, hypoxia caused a decrease of pPE in human pulmonary arterial endothelial cells (HPAEC), while supplementation of plasmalogens protected HPAEC from hypoxia^19^. As described above, pPE (38:5) and pPE (38:4) may be composed of AA, so these lipids could be storage sites for inflammatory lipid mediators in EC^37^. Because EC lining blood vessels are exposed to oxidative stress, an increase of plasmalogens might protect these cells against such stress.

SM levels declined transiently during differentiation of iPSC to MC and EC. SM accounts for about 10% of total lipids in HUVEC^38^. Sphingosine-1-phosphate (S1P) is a lipid mediator formed from SM that has an important role in embryonic angiogenesis^45^. SM is a basic component of cell membranes, and is a major source of ceramides regulated by SMS and SMases, as described above^25^. Inhibition of SMS was found to enhance the barrier function of EC via changes of the actin cytoskeleton that resulted in reduction of the loss of SM from lipid rafts in cell membranes^27^. In the present study, changes of cell morphology were noted during differentiation, suggesting that dynamic changes of the actin cytoskeleton occur with differentiation. The transient decrease of SM might have occurred because it is metabolized and consumed during differentiation. A previous study showed that SM (34:2) was lower in murine iPSC than murine fibroblasts^36^, and the present study also demonstrated that the levels of most SM components were lower in iPSC than ECpa. When the SM levels in ECpa were compared to those in HUVEC (endothelial cells) and HFL-1 cells (fibroblasts), most SM levels were lower in ECpa than HUVEC, except for SM (36:0) and SM (36:1), while the SM levels of ECpa varied in comparison to those of HFL-1 cells. From these findings, SM levels in cells vary among different cell types. SM (34:0) was lower in ECpa than HFL-1. HFL-1 was cultured by F12K^®^ medium and F12K^®^ contained much of SM (34:0) as shown Supplementary Data Fig. S6. SM(34:0) in F12K medium might have had a influence of SM(34:0) in HFL-1. In short, SM might be consumed during differentiation and possibly contributes to differentiation from iPSC to EC.

In a clinical study, high plasma SM levels were markers for more rapid annual progression of pulmonary emphysema^26^, and elevation of plasma SM was observed in patients with cystic fibrosis^46^. SM levels also increase in patient with acute coronary syndrome^42^. These data suggest that SM could be a marker for tissue remodeling or acute EC injury, but further study of the changes of SM in EC is needed.

There were several limitations of present study. First, we only examined the levels of plasmalogens and SM. However, to reveal the entire lipid metabolic signature of EC, it would be necessary to also investigate other lipids, such as PC, PE, phosphatidylserines (PS), and lysophosphatidic acids (LPA). Although there are no simple and established methods for induction of cells other than EC from iPSC, evaluating the changes of pPE levels during differentiation or in response to various stimuli when compared with other cells derived from iPSC or iPSC generated from different resources could help to determine the physiological significance of pPE in ECpa. Furthermore, we employed co-culture of iPSC-derived EC and HFL-1 cells, but EC derived from iPSC are not necessarily the same as native EC. When HUVEC were cultured until the 14th passage, oxidized LDL levels, tumor necrosis factor-alpha-induced apoptosis, and caspase-3 activity were all significantly enhanced by more than 3-fold compared with young cells in the 3^rd^ passage^47^. The metabolic signature of senescence in donor iPSC might affect generation of EC from iPSC^48, 49^. Therefore, to evaluate the usefulness of the present co-culture system, it would be better to examine the metabolic signature of EC senescence by longer passaging.

In conclusion, metabolism of plasmalogens (especially pPE) and SM showed various changes during differentiation of iPSC to EC and subsequent culture of EC phenotype acquisition. This is the first report about the changes of plasmalogens during differentiation of iPSC to EC. pPE (38:5) and pPE (38:4) could be markers for mature EC, while a decrease of SM is a transient marker for MC and immature EC. These lipids may be associated with differentiation of iPSC to EC, but further analysis is needed to elucidate their functional significance.

## Materials and Methods

### Cell culture

Human iPSC were purchased from RIKEN-BRC (409B2, obtained from a healthy 36-year-old woman), and the mouse embryonic fibroblasts cell line (SNL76/7) was purchased from the European Collection of Authenticated Cell Cultures (ECACC)^50^. Human umbilical vein endothelial cells (HUVEC) and human fetal lung fibroblast-1 (HFL-1) cells were obtained from the American Type Culture Collection (ATCC). The following media were used for cell culture: Primate ES Cell medium^®^ (ReproCell) with 4 ng/mL bFGF (ReproCell) for feeder iPS culture, StemFit^®^ (ReproCell) for feeder-free iPSC culture, Dulbecco’s modified Eagle’s medium^®^ (DMEM, Nacalai Co. Ltd., Japan) with 7% fetal bovine serum and penicillin-streptomycin for SNL76/7 cells, VEGF Comp Kit^®^ medium for HUVEC and EC derived from iPSC, and Ham’s F-12K (Kaighn’s)^®^ medium (Thermo Fisher Scientific) with 10% fetal bovine serum albumin (BSA) and penicillin-streptomycin (1:100, Invitrogen) for HFL-1 cells. Human plasma fibronectin-coated plates/dishes (3 μg/cm^2^, Gibco) were used for EC culture. Type I collagen pre-coated plates (Iwaki Co. Ltd., Japan) were also used for culture of EC derived from iPSC. Culture of iPSC was performed as reported previously^50–52^, as was feeder-free iPSC culture. In brief, iPSC were removed from feeder culture dishes by incubation with CTK solution^®^ (ReproCell) for 1–2 min at 37 °C. Then the cells were washed with phosphate-buffered saline (PBS) and incubated with 0.5 × TrypLESelect^®^ (Thermo Fisher Scientific) for 4–7 minutes. After removing TrypLESelect^®^, the cells were washed with PBS and scraped off the culture dish for sub-culture on dishes coated with laminin-511 E8 (iMatrix-511^®^, ReproCell). StemFit^®^ (ReproCell) medium was used for feeder-free iPSC culture.

### Induction of differentiation

Induction of differentiation of iPSC to EC was done by a modification of the previously reported method^30, 31^. Feeder-free iPSC were subcultured in 6-well plates at a density of 0.2 × 10^5^ cells/well on day −2. Differentiation induction medium was prepared with Advanced RPMI 1640^®^ (Thermo Fisher), 2% B-27 Minus Insulin^®^ (Gibco), 200 mM L-Glutamine (Gibco), 10 ng/mL of fibroblast growth factor (FGF2, ReproCell), 50 ng/mL of VEGF A-165 (Wako Co. Ltd., Japan), and 2 ng/mL of bone morphogenetic protein 4 (BMP-4, R&D). The standard culture medium was changed to induction medium with 1% matrigel (Corning) on day 0. Induction medium was exchanged every two days, and differentiation was completed on day 5 (Fig. 1). After sorting of EC (iEC) and MC by FACS analysis, only iEC were subjected to following subculture.

### Cell sorting and flow cytometric analysis

Differentiated cells were detached by incubation with ACCUMAX^®^ (Innovative Cell Technologies) for 15 min at 37 °C. Detached cells were sorted by using a FACS Aria^®^ (BD) with anti-CD309-PE (VEGFR-2/KDR, Milteny) and anti-human CD144 (VE-Cadherin)-APC (eBioscience). Sorted cells were suspended in EC medium and incubated on type I collagen-coated culture plates. PE mouse IgG1κ isotype control (BD) and APC Mouse IgG1κ isotype control (BD) antibodies were used in this experiment. For confirmation of ECpa purification efficiency, anti-human CD31 (PECAM-1)-FITC (eBioscience) and FITC mouse IgG1 isotype control (BD) were also used.

### Immunostaining

Culture plates were fixed with 4% paraformaldehyde for 15 min at room temperature and rinsed with PBS. Cells were permeabilized by addition of chilled methanol for 10 min at −20 °C, followed by washing three times with PBS and incubation with 1% BSA for 1 hour. After washing three times with PBS again, cells were incubated with the primary antibody at the recommended concentration according to the manufacturer’s instructions. Human CD31/platelet endothelial cell adhesion molecule-1 (PECAM-1) antibody (R&D), human VE-cadherin antibody (R&D), VEGFR2 antibody (Invitrogen) and human von Willebrand Factor (vWF) antibody (Abcam) were used as the primary antibodies. After incubation, cells were washed three times with PBS and then incubated for 1 hour with the secondary antibody (1:50), which was goat anti-rabbit IgG-Alexa Fluor 555 (Invitrogen) or goat anti-mouse IgG-Alexa Fluor 488 (Invitrogen). Nuclear staining was performed with 4′,6-diamidino-2-phenylindole dihydrochloride (DAPI, Invitrogen). Since iEC were purified EC by FACS sorting, the expression of vWF protein were done on cytospin samples (Auto Smear 120, SAKURA, Tokyo, Japan).

### Real time quantitative RT-PCR analysis

The assay of quantification of mRNA levels of iPSC and iPSC-derived endothelial phenotype cells was carried out after isolation of total RNA (TRIzol^®^ reagent, Thermo Fisher) using Takara PCR Thermal Cycler MP (TP3000) as described previously^53^. The sequences of primers used for amplification of vWF and GAPDH were follows; vWF forward gaaatgtgtcaggagcgatg, reverse atccaggagctgtccctca, GAPDH forward tcaacagcgacacccactcc, reverse tgaggtccaccaccctgttg.

### Tube formation assay

Before the tube formation assay, 12-well dishes were pre-incubated with 200 μL of extracellular matrix for 1 hour at 37 °C. Then passaged EC (ECpa) from passages 2 to 5 derived from iPSC culture and sorted (iEC) were incubated on the matrix for 2 to 6 hrs at 0.5 × 10^5^ cells/well. The assay was performed with an endothelial Tube Formation Assay^®^ kit (Cell Biolabs).

### Co-culture network formation assay

This assay was done by a modification of the reported method^54^. HFL-1 cells were seeded in multi-well plates with F-12K medium at 1.5 × 10^5^ cell/cm^2^ and were incubated for 5 days. Then ECpa were seeded onto the HFL-I culture dishes at 1.5 × 10^5^ cell/cm^2^ with a VEGF Comp Kit^®^. Incubation was continued until 7 days and the EC network assay was performed on day 7.

### Purification of lipids

Total lipids were extracted from cells by the Bligh-Dyer method as reported previously^50, 55^. Briefly, cells were washed twice with normal saline, harvested with a scraper, and sonicated before measurement of the protein concentration. Then the protein solution (100 μg) was mixed with methanol and chloroform in a glass tube (solution: methanol: chloroform = 900 μL: 2 mL: 1 mL). The culture medium (10 μl) was mixed with the methanol and chloroform in a glass tube, and lipids from each medium used for cell culture were also extracted as the same manner. The following internal standards for lipids (1 μg) were obtained from Avanti Polar Lipids (Alabaster, AL, USA): PC (30:0, 15:0/15:0), PE (34:0, 17:0/17:0), and SM (35:0, dihydroxy (d)18:1/17:0).

### LC-MS/MS

Lipids were analyzed by reverse-phase ultra-high-pressure liquid chromatography (UHPLC) using an Acquity UPLC BEH C18 column^®^ (1.7 μm, 2.1 × 50 mm) (Waters, MA, USA) coupled to a 5500 QTRAP^®^ mass spectrometer (Sciex Inc., MA, USA), as previously reported^50^. A binary gradient was used, which consisted of solvent A (acetonitrile:methanol:water (1:1:3) containing 5 mM ammonium acetate) and solvent B (2-propanol containing 5 mM ammonium acetate) with the following gradient profile: 0–1 min, 95% A; 1–9 min, 5–95% B linear gradient; 9–13 min, 95% B. The flow rate was 0.3 ml/min and the column temperature was 40 °C. The following settings were employed: source voltage 5000 and −4500 V for positive and negative, respectively; GS1 50; GS2 80; curtain gas 30; and source temperature 100 °C. Multiple reaction monitoring (MRM) of the transitions of individual lipids was done as described previously^56^. Individual lipid species were normalized against the internal standards, which were PC (30:0, 15:0/15:0), PE (34:0, 17:0/17:0), and SM (35:0, d18:1/17:0).

### Statistical analysis

The reported results are data from at least three independent experiments. Mean values and the standard error of the mean (SEM) were calculated. Student’s *t*-test was used for assessment of significant differences and analyses were performed with 4 step Excel tokei^®^ software (Seiunsya, 2015, Japan).

## Electronic supplementary material

Supplementary Information
