# Supplementary material for: Changes of plasmalogen phospholipid levels during differentiation of induced pluripotent stem cells 409B2 to endothelial phenotype cells
Source: Sci Rep. 2017 Aug 24;7:9377. doi: 10.1038/s41598-017-09980-x (PMC5571164; doi:10.1038/s41598-017-09980-x)
Supplement: Supplementary file 1 — Supplementary Information [file 41598_2017_9980_MOESM1_ESM.pdf]

**Title: Changes of plasmalogen phospholipid levels during differentiation of induced pluripotent stem cells 409B2 to endothelial phenotype cells**

Yusuke Nakamura[1], Yasuo Shimizu[1,*] Yasuhiro Horibata[2], Rinna Tei[1], Ryosuke Koike[1], Meitetsu Masawa[1], Taiji Watanabe[1], Taichi Shiobara[1], Ryo Arai[1], Kazuyuki Chibana[1], Akihiro Takemasa[1], Hiroyuki Sugimoto[2], and Yoshiki Ishii[1]

[1]Department of Pulmonary Medicine and Clinical Immunology, [2]Department of Biochemistry, Dokkyo Medical University School of Medicine, 880 Kitakobayashi, Mibu, Tochigi 321-0293, Japan.

**Supplementary data 1**

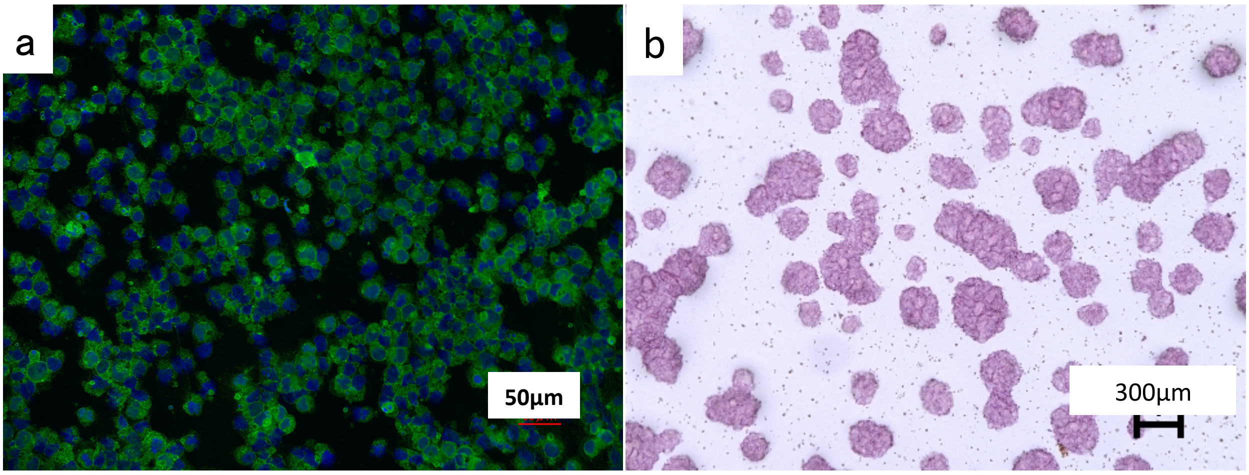


**Figure S1 | Confirmation of pluripotency of feeder-free iPSC.** SSEA-4 immunofluorescence staining assay (a). Immunofluorescence staining was done with SSEA-4 Antibody (Santa Cruz) for 1st antibody and Goat anti-Rabbit IgG-Alexa Fluor 555(Invitrogen) for 2nd antibody (green: SSEA-4 and blue: DAPI). Alkaline Phosphatase assay (b). iPSC were treated with 4% paraformaldehyde (PFA) for 10 minutes. Then, washed with ultrapure water three times. Following step was done by using alkaline Phosphatase kit® according to manufacturer's instruction (Sigma-Aldrich).

**Supplementary data 2**

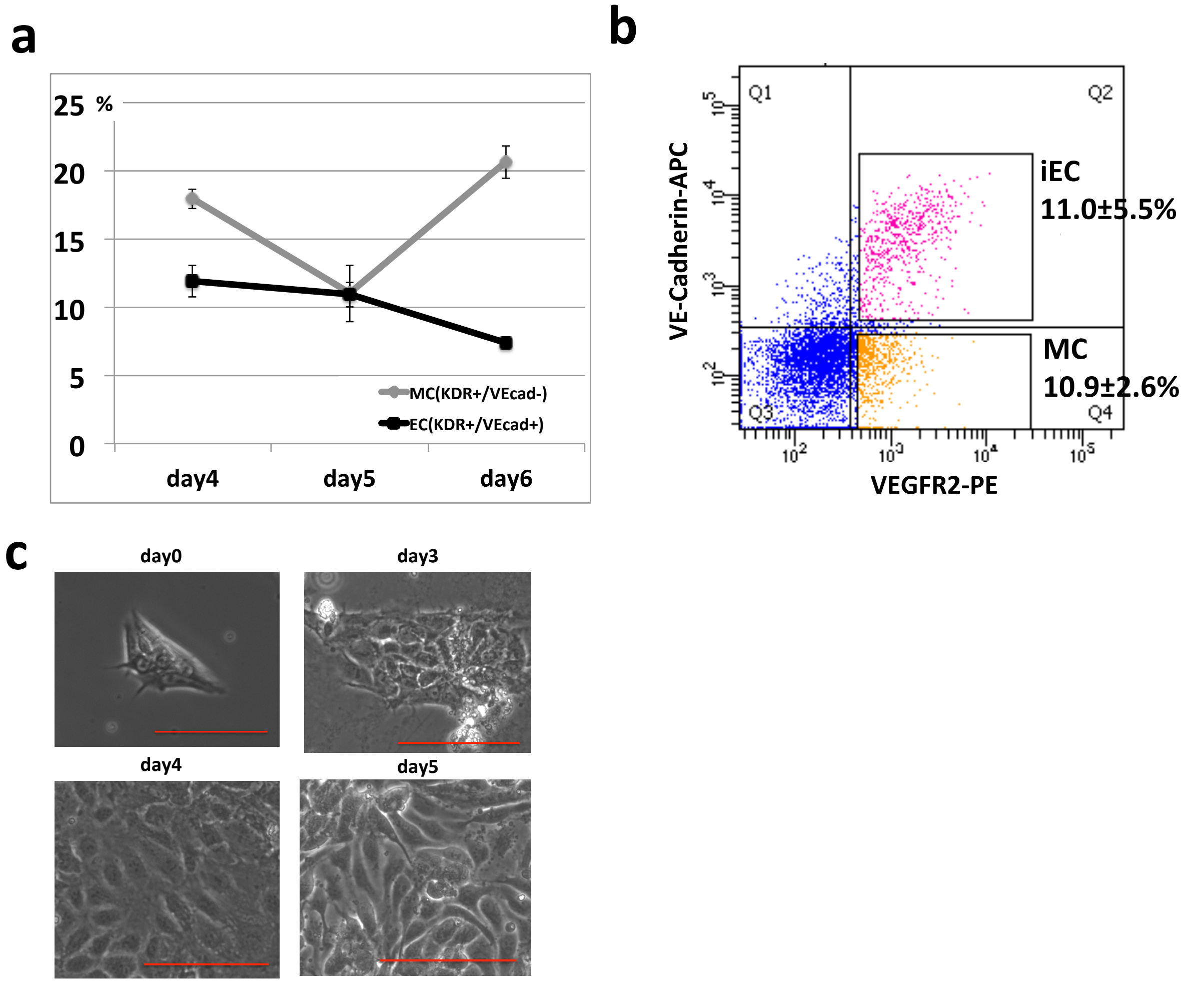


**Figure S2 | Morphological changes and differentiation efficiency during differentiation.** Ratios of MC (KDR(VEGFR2R)$^{+}$/VE-cadherin$^{-}$) and iEC (KDR(VEGFR2R)$^{+}$/VE-cadherin$^{+}$) differentiating from iPSC (a). The differentiation experiment was conducted at least 5 times. Sorting efficacy of MC (10.9 ± 2.6 %) and iEC (11.0 ± 5.5 %) on day 5 by FACS analysis (b). Morphological changes of iEC during differentiation from feeder-free iPSC (c). iPSC were stimulated by cytokines on day 0. iPSC characteristically had large nuclei and scanty cytoplasm, but these features changed from days 3 to 4. Differentiated cells were purified on day 5 in five independent experiments. *(Scale bar = 100 µm)*.

**Supplementary data 3**

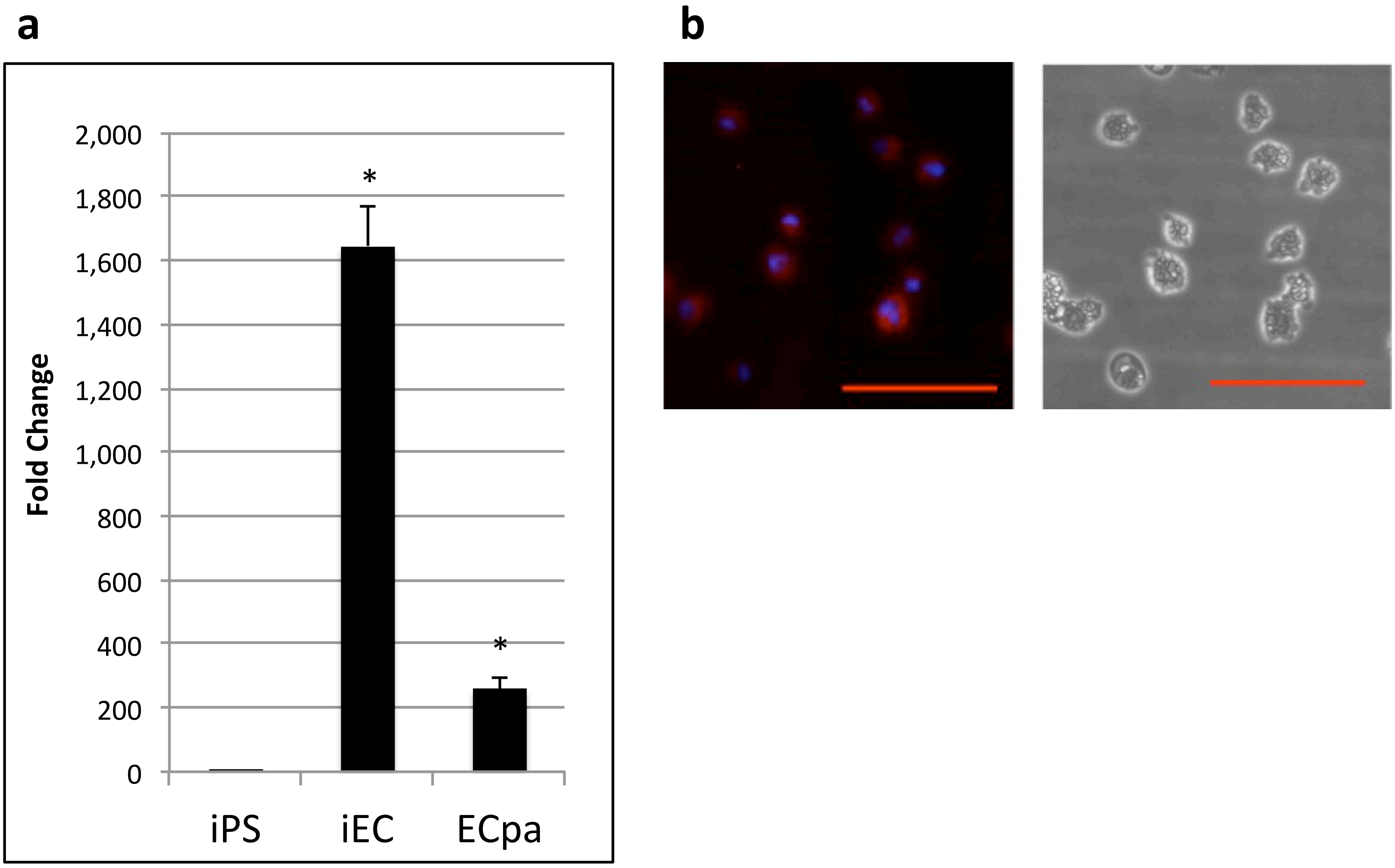


**Figure S3 | Quantitative RT-PCR analysis for the expression of von Willebrand factor.** vWF expression was measured in iPSC, iEC and ECpa by RT-PCR. These expressions were shown with iPSC vWF/GAPDH fold change (a). (*$p$<0.05 compared with iPSC). Immunostain for iEC (b). Left panel shows vWF positive iEC (red: vWF, blue: DAPI) and right panel shows bright field. (*Scale bar = 100 μm*).

**Supplementary data 4**

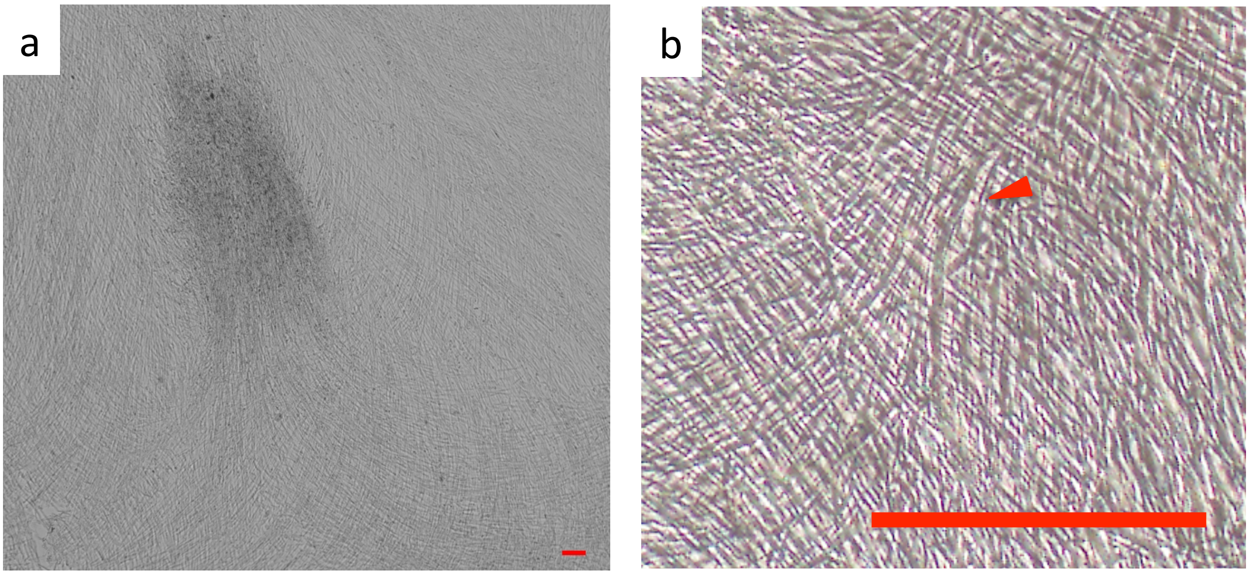


**Figure S4 | Co-culture experiment for ECpa and HFL-1.** ECpa was co-cultured with HFL-1 as described in material and method. Bright field image (a). Magnified figure of bright field, arrowhead indicates tube formations on bright field (b). Networks were difficult to distinguish from HFL-1 to ECpa without immunostaining (*Scale bar=100μm*).

**Supplementary data 5**

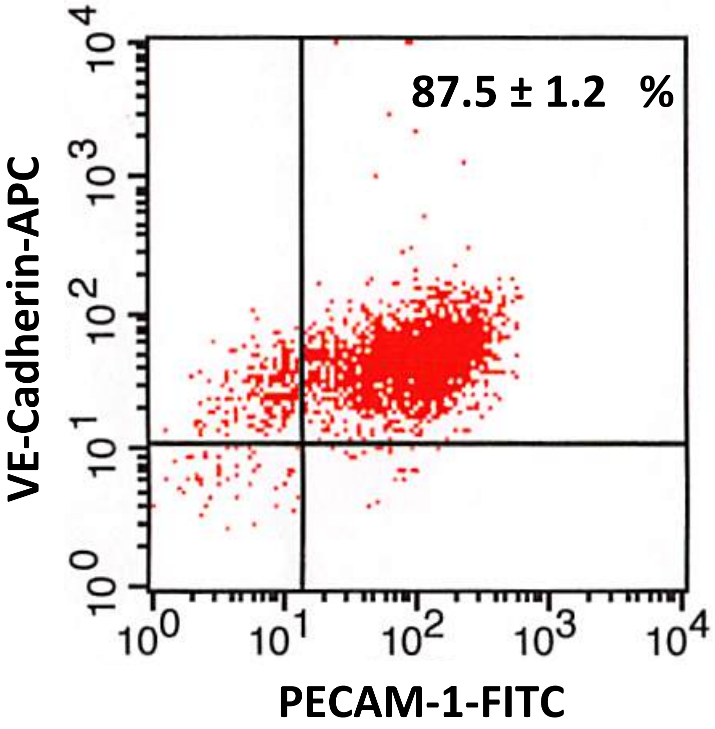


**Figure S5 | VE-cadherin$^+$/ PECAM-1$^+$ ratio of ECpa.** VE-cadherin / PECAM1 positive ratio of ECpa was nearly 90%.

**Supplementary data 6**

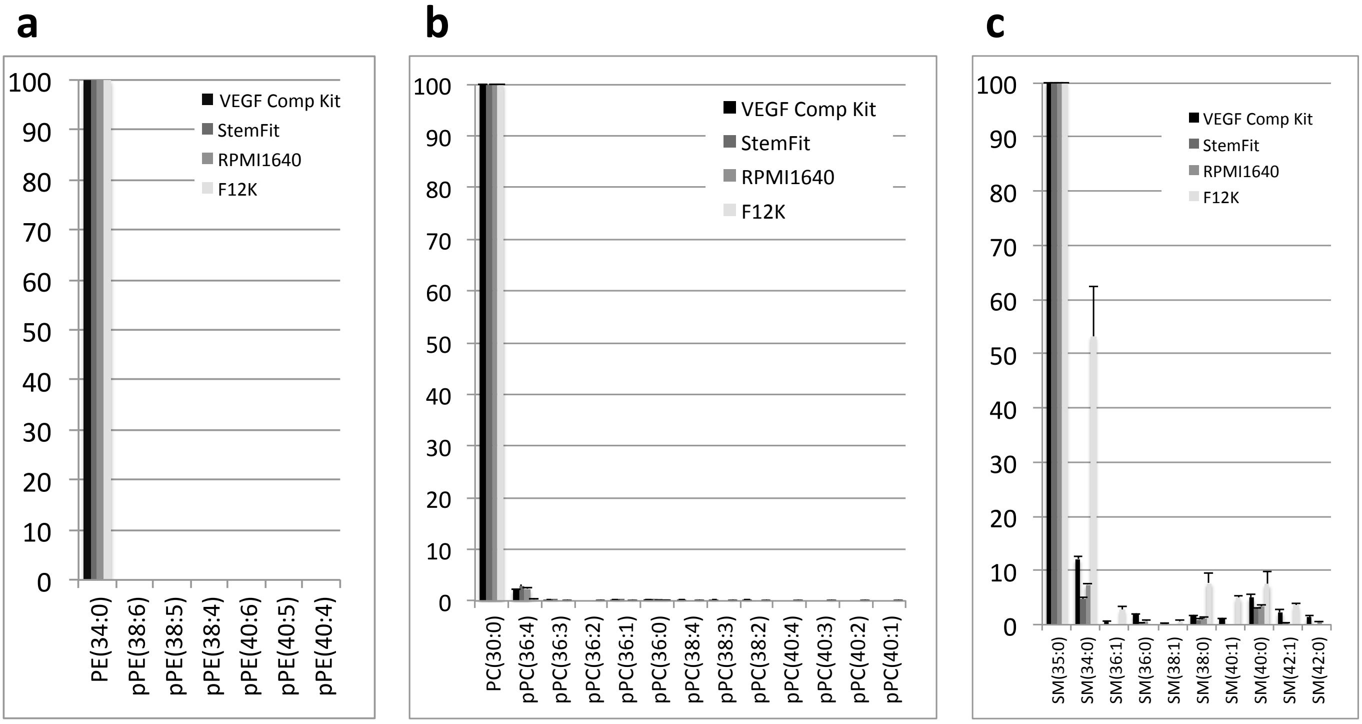


**Figure S6 | Phospholipids examined by LC-MS/MS of culture medium.** Culture medium of VEGF comp kit® for iEC, ECpa and HUVEC, Stemfit® for feeder-free iPSC, RPMI 1640® used in induction of differentiation for iPSC and Ham's F-12K® for HFL-1 were examined. Most of pPE, pPC were nearly under detection levels, however the levels of SM (34:0) in Ham's F-12K® was high.

## Supplementary data 7

**a**

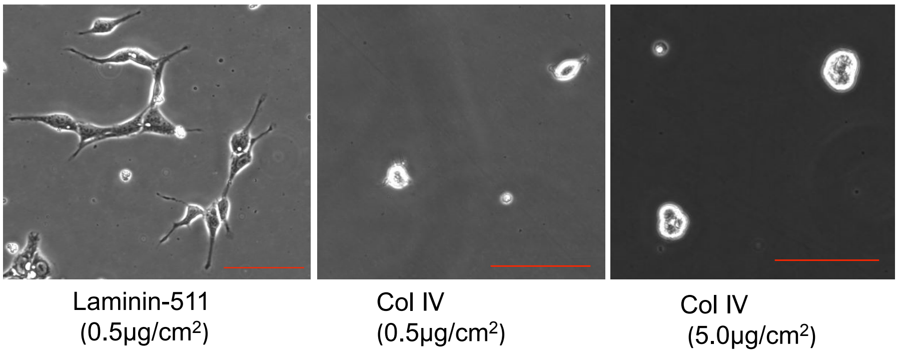


**b**

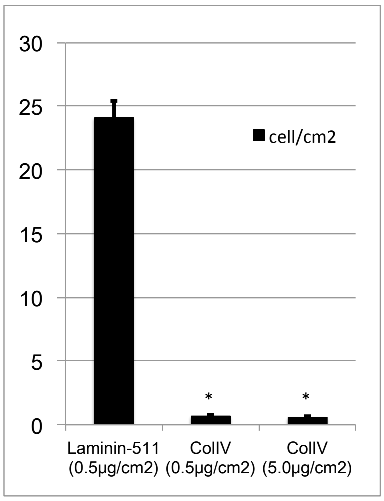


**Figure S7 | The efficacy of adhesiveness of feeder-free iPSC.** Feeder-free iPSC ($1\times10^4$ cells) was cultured on 12 well plate coated with Laminin-511 (0.5µg/cm$^2$), type IV Collagen (Col IV) (0.5µg/cm$^2$) and Col IV (5µg/cm$^2$) for 3 hours at 37°C incubator, respectively. Attached cell was counted after 24 hours incubation. Round morphological changes was seen on Col IV wells (a). Cell adhesiveness of Laminin-511 coated culture dish showed greater (24.1 ± 1.3 cell/cm$^2$) than Col IV coated culture dish (0.6 ± 1.3 cell/cm$^2$ at the concentration of 0.5µg/ml, or 0.7 ± 0.1 cell/cm$^2$ at the concentration of 5.0 µg/ml, mean ± SEM)(b). (*Scale bar=100µm*) (*$p<0.05$ compared with Laminin-511) .
